# Impact of cardiovascular involvement on the clinical course of paediatric mitochondrial disorders

**DOI:** 10.1186/s13023-020-01466-w

**Published:** 2020-07-31

**Authors:** Alice Brambilla, Iacopo Olivotto, Silvia Favilli, Gaia Spaziani, Silvia Passantino, Elena Procopio, Amelia Morrone, Maria Alice Donati

**Affiliations:** 1 Paediatric Cardiology Unit, Anna Meyer Children Hospital, Viale Pieraccini 24, 50139 Florence, Italy; 2 Inborn Metabolic and Muscular Disorders Unit, Anna Meyer Children Hospital, Florence, Italy; 3 Cardiomyopathy Unit, Careggi University Hospital, Florence, Italy

**Keywords:** Cardiomyopathy, Mitochondria, Paediatrics, Heart failure

## Abstract

**Background:**

Primary mitochondrial disorders (PMD) are rare conditions resulting in progressive multi-organ failure. Cardiovascular involvement (CVI) has been reported in paediatric patients. However, its age-related prevalence, clinical presentation and prognostic impact are unresolved. We detailed CVI in a cohort of children diagnosed with PMD over two decades at a tertiary referral centre.

**Results:**

We enrolled 86 PMD patients (M/F = 30/56; mean age 6.4 ± 8.58 years). CVI was detected in 31 patients (36%), with mean age at onset of 5.7 ± 7.8 years including the pre- and neonatal phase in 14, often representing the first sign of PMD (42% of those with CVI). Heart disease resulted more common in males and in children with specific aetiologies (Barth, TMEM70 and MELAS syndromes). Hypertrophic, non-compaction and dilated cardiomyopathies were the prevalent disorders, although pulmonary arterial hypertension was also found. Adverse cardiac events (heart failure, resuscitated cardiac arrest, ICD/PM implantation, sudden death) occurred in 19% of children with CVI over a follow-up period of 5.4 ± 4.3 years. All-cause mortality was higher in patients with CVI compared to those without CVI (45.1% vs 21.8%; *p* < 0.01); female sex, age at onset < 5 years, acute heart failure at presentation and diabetes also proved independent predictors of outcome.

**Conclusion:**

Cardiovascular involvement occurred in over one-third of children diagnosed with PMD, often at a very early age, and was associated with adverse prognosis. Final outcome of PMD-related CVI was influenced by the specific underlying aetiology, suggesting the need for tailored management of heart failure and sudden death prevention.

## Background

Primary mitochondrial disorders (PMD) comprise a group of rare genetic disorders characterized by impaired energy production due to mutations in nuclear or mitochondrial genes involved in oxidative phosphorylation. Organs with high-energy requirements, such as brain, heart and skeletal muscle, rely heavily on mitochondrial function, are more severely affected [1].

Besides the defects in mitochondrial proteins, the impairment of the regulatory pathways at the basis of mitochondria homeostasis may lead to mitochondrial dysfunction, decreased autophagy and neuronal death [2]. These mechanisms have been proved to be causative of distinct neurodegenerative disorders and age-related pathologies, other than being potentially involved in the maturation of cardiomyocytes [3]. Inducers of mitophagy, the process that selectively removes damaged or redundant mitochondria, are currently under investigation as potential therapeutic strategy for mitochondrial related disorders [4].

Cardiovascular involvement (CVI) has been described in PMD [5, 6]); however, its age-related prevalence, clinical presentation and prognostic impact are still incompletely resolved. Specifically, whether the outcome of CVI is comparable across the spectrum of PMD is difficult to assess due to the rarity and heterogeneity of the different subsets. The issue bears relevance to individual management of these children who are categorized under the same diagnostic umbrella but may have radically different phenotypes and clinical outcome. Aim of this study was to assess CVI over long-term follow-up in a sizeable cohort of children with PMD extensively characterized and followed over the last two decades at a tertiary referral centre for metabolic disease.

## Methods

We retrospectively analysed clinical, biochemical, histological and genetic records of 86 children diagnosed with PMD, consecutively evaluated at Anna Meyer Children Hospital from January 1997 to December 2017. The investigation conforms to the principles outlined in the Declaration of Helsinki. Local paediatric ethics committee at Anna Meyer Children Hospital has approved the study protocol; written informed consent forms have been collected from living patients or their relatives.

### Diagnosis of primary mitochondrial disorder

Over the last two decades, 365 children have been evaluated for suspected primary mitochondrial disorders: in 86, PMD was diagnosed based on the Modified Walker Criteria [7]. Clinical and laboratory assessment at baseline is described in Table 1. Patients presenting characteristic phenotypes (i.e. MELAS syndrome, TMEM70 deficiency, Barth syndrome) were referred for molecular investigation. In the absence of evocative phenotype, muscular biopsy for standard histology/ histochemistry, electron microscopy and mitochondrial enzyme assay was performed. The standard diagnostic panel included hematoxylin and eosin, modified Gomori trichrome, lipid stains, succinate dehydrogenase (SDH), and cytochrome oxidase enzyme histo-chemistry. Biochemical assays of respiratory chain complex activity were systematically performed, whereas electron microscopy was added only in selected cases. If the muscular biopsy was consistent with underlying mitochondrial disorder, mt-DNA analysis was performed on leukocytes, myocytes or urinary epithelium. Large deletions-duplications were documented by Southern Blot or Next Generation Sequencing. Quantitative real-time PCR quantification of total mt-DNA content in affected tissues was required to confirm mitochondrial DNA depletion syndromes; a reduction in mt-DNA copy number > 60–65% of the average recorded in age-matched controls confirmed the diagnosis. In case of negative results despite high clinical/biochemical suspicion, exome sequencing was considered.

**Table 1:** Baseline and follow-up assessment of patients diagnosed with Primary Mitochondrial Disorders

Family history	• Pattern of transmission (autosomal, X-linked, matrilineal) • Recurrence of distinctive features:
	- Myopathy
	- Mental retardation/epilepsy
	- Precocious death/stillbirth
	- Diabetes
	- Hearing loss
	- Cardiovascular diseases
	- Short stature
	- Ocular involvement.
Clinical examination	• Auxological parameters (weight, length/stature, cranial circumference) • Vital parameters (blood pressure, heart rate, respiratory rate, oxygen saturation) • Assessment of neurological status and cognitive development
Specialist evaluations	• Audiological screening • Ophthalmologic screening in order to exclude: - retinitis
	- cataract
	- optic nerve atrophy
	- progressive loss of vision
Cardiologic evaluation	• Clinical examination • 12-lead ECG and transthoracic echocardiograph
Brain Magnetic Resonance	• Common findings:
	- Leigh syndrome
	- Focal hypodense lesions
	- Cortical and cerebellar atrophy
	- White matter hyperintensity
	- Basal ganglia involvement
	- Agenesis/hypoplasia of the corpus callosum
Brain MR spectroscopy	Abnormal accumulation of lactate in the parenchyma and cerebrospinal fluid
Laboratory investigation	• Complete blood count • Liver and renal functional tests • Blood fasting glucose • Blood gas analysis • Plasma lactate levels • Creatine phosphokinase (CPK) • Plasma amino acids • Urine amino and organic acids • Redox status • Acylcarnitine profiles

Eight patients with paediatric onset Mitochondrial myopathy, encephalopathy, lactic acidosis, and stroke (MELAS) syndrome from the present cohort have been included in a recent publication addressing cardiac phenotypes [8] Four patients diagnosed by whole exome sequencing have been described as isolated case reports [9–11].

### Definition of cardiovascular involvement

All patients were screened for CVI at initial evaluation, including complete clinical examination, twelve-lead ECGs and transthoracic echocardiography. M-mode measurements were indexed to age and body-surface area and corresponding z-scores were derived. Further investigations were added as appropriate in patients with CI, according to clinical status (24-h Holter monitoring, cardiac catheterization, cardiac magnetic resonance, exercise test).

Cardiovascular involvement associated with PMD was defined by one of the following:

- Hypertrophic cardiomyopathy (HCM): septal or left ventricular (LV) posterior wall thickness over 2 Z score, in absence of other causative conditions;
- Dilated cardiomyopathy (DCM): increased ventricular end diastolic inner diameter (> 2 Z Score) occurring with systolic dysfunction, assessed on the basis of ejection fraction (EF). EF was measured trough biplane Simpson method (normal value > 55%, mild dysfunction 45–54%, moderate dysfunction 30–44%, severe dysfunction < 30%).
- Non-compaction (NC) phenotype: hyper-trabeculated aspect of left ventricular walls with end systolic ratio of non-compacted to compacted layers over 2; it was considered a pathologic finding only if associated with previous phenotypes or in presence of ventricular dysfunction.
- Pulmonary arterial hypertension (PAH): increase in mean pulmonary arterial pressure (PAPm) ≥25 mmHg at rest as assessed by right heart catheterization
- Arrhythmic disorders and high-grade atrioventricular (AV) block (type II second-degree AV block, third-degree AV block)

### Follow-up protocol and study end-points

After initial evaluation, follow-up visits were scheduled at least every 6 months, including clinical examination, neurologic evaluation and metabolic assessment [Table 1]. Cardiologic follow-up was planned every 3–6 months in the presence of CVI, otherwise every 12 months.

Overt CVI involvement was defined in presence of clinical signs and/or symptoms (dyspnoea, tachycardia, hypo- or hypertension, gallop rhythm, cyanosis, hypoxemia, excessive swelling, feeding difficulties or fatigue not attributable to neurologic/muscular impairment, pathologic murmur).

Twenty-four hour Holter monitoring was proposed in case of pathologic ECG at rest, or in presence of signs/symptoms suggestive for rhythm anomalies. Right heart catheterization was performed only in patients with echocardiographic evidence of pulmonary hypertension.

Major adverse cardiac events (MACEs) were recorded, including hospitalization for heart failure (HF), death due to HF, sudden death, resuscitated cardiac arrest, high-grade AV block, life-threatening arrhythmias and cardiac transplantation. All cause and cardiac mortality were assessed.

### Statistical analysis

Continuous variables were presented as mean ± standard deviations and discrete variables are presented as counts and percentages. Multivariate regression models (Probit) were used to compare patient subsets with and without CVI. All tests were two-tailed, and *P*-values less than 0.05 were considered significant. Kaplan Meier curves were used to compare survival between the two groups (with and without CVI) and to show MACE-free survival in CVI group. The STATA statistical software version 15 was used for all analyses.

## Results

### Genetic spectrum of PMD

The study enrolled 86 patients (30 males/56 females) with established diagnosis of PMD. Mean age at diagnosis was 6.4 ± 8.6 years; in 3/83 patients (3.6%) the diagnosis was confirmed post-mortem. Muscular biopsy was performed in 55 (64%) patients; of these, 27 showed combined defects, with deficit in I-IV (11%) and I-III-IV (11%) complexes being the most frequent [Table 2].

**Table 2:** Clinical features, muscular biopsy, molecular diagnosis and outcome in enrolled patients

	Overall	Patients without CVI	Patients with CVI	***p***-value
Number of patients (%)	86	55 (64%)	31 (36%)	
Males/Females	0.5	0.15	1.8	< 0.01
Mean age at diagnosis (years; ±SDS)	6.4 (±8.58)	7.8 (±10.08)	5.75 (±7.67)	0.3
Mean age at LFUP (years; ±SDS)	11.4 (±10.1)	11.8 (±9.17)	10.98 (±11.5)	0.7
Muscular biopsy (n° patients; %)	55 (63.9%)	37 (67.2%)	18 (58%)	0.4
Respiratory chain enzyme deficiency (n°;%)	49 (57%)	35 (63.6%)	14 (45%)	
- Complex I	12 (14%)	9 (16.3%)	3 (9.7%)	0.7
- Complex II	1 (1.2%)	1 (1.8%)	–	0.5
- Complex III	2 (2.3%)	2 (3.6%)	–	0.4
- Complex IV	3 (3.5%)	2 (3.6%)	1 (3.2%)	0.8
- Complex V	4 (4.6%)	2 (3.6%)	2 (6.4%)	0.3
- Combined deficiencies	27 (31.4%)	19 (34.5%)	8 (25.8%)	0.8
Molecular analysis (n°;%)	57 (66.3%)	31 (56.3%)	26 (83.8%)	
- MTTL1 (A3243G)	8 (9.3%)	3 (5.4%)	5 (16.1%)	0.4
- TMEM70	5 (5.8%)	–	5 (16.1%)	< 0.01
- NARP	4 (4.6%)	3 (5.4%)	1 (3.2%)	0.4
- Barth syndrome	4 (4.6%)	–	4 (12.9%)	0.02
- POLG	2 (2.3%)	2 (3.6%)	–	0.4
- ACAD9	2 (2.3%)	1 (1.8%)	1 (3.2%)	0.4
- SURF1	1 (1.2%)	1 (1.8%)	–	0.4
- NDFUS1	1 (1.2%)	–	1 (3.2%)	0.8
- SDHB	1 (1.2%)	1 (1.8%)	–	0.4
- ELAC2	1 (1.2%)	–	1 (3.2%)	0.4
- DGUOK	1 (1.2%)	1 (1.8%)	–	0.4
- TK2	1 (1.2%)	1 (1.8%)	–	0.4
- tRNA (Glu)	1 (1.2%)	–	1 (1.2%)	0.4
- NAHD dehydrogenase (complex I) subunits:	7 (8.1%)	5 (9%)	2 (6.5%)	0.07
o MTND1	2 (2.3%)	1 (1.8%)	1 (3.2%)	
o MTND2	1 (1.2%)	1 (1.8%)	–	
o MTND3	1 (1.2%)	–	1 (3.2%)	
o MTND5	1 (1.2%)	1 (1.8%)	–	
o MTND6	2 (2.3%)	2 (3.6%)	–	
- MTCYB1	1 (1.2%)	1 (1.8%)	–	0.4
- MT-CO1	1 (1.2%)	1 (1.8%)	–	0.4
- MT-TC	1 (1.2%)	1 (1.8%)	–	0.4
- Leber’s hereditary optic neuropathy	1 (1.2%)	1 (1.8%)	–	0.4
- Kearns-Sayre disease	2 (2.3%)	1 (1.8%)	1 (3.2%)	0.8
- Sengers disease	1 (1.2%)	–	1 (3.2%)	0.2
- Large mt-DNA depletion	2 (2.3%)	1 (1.8%)	1 (3.2%)	0.8
- TIMMDC1	1 (1.2%)	1 (1.8%)	–	0.4
- NUBPL	1 (1.2%)	1 (1.8%)	–	0.4
- SFXN4	1 (1.2%)	1 (1.8%)	–	0.4
- ATAD3A	1 (1.2%)	–	1 (3.2%)	0.2
- DARS2	1 (1.2%)	1 (1.8%)	–	0.4
- SLC19A3	1 (1.2%)	–	1 (3.2%)	0.2
Extra-cardiac manifestations (n°; %)				
- Failure to thrive	28 (32.5%)	12 (21.8%)	16 (51%)	0.1
- Neurologic	52 (60.4%)	33 (60%)	19 (52.7%)	0.9
o neurodevelopmental delay	36 (42%)	21 (38%)	15 (48%)	–
o epilepsy	28 (32.5%)	14 (25.5%)	4 (13%)	–
o hypo- or hypertonia	17 (19.7%)	7 (12.7%)	10 (32%)	–
o recurrent headache/cerebral stroke	6 (7%)	3 (5.5%)	3 (9.7%)	- -
- Ocular	16 (18.6%)	6 (11%)	10 (32%)	0.1
- Hearing loss	8 (9.3%)	2 (3.6%)	6 (19.3%)	0.3
- Diabetes	5 (5.8%)	1 (1.8%)	4 (13%)	0.1
- Renal failure	6 (7%)	1 (1.8%)	−5 (16%)	0.04
Cardiac involvement	31 (36%)	–	31 (100%)	n.a.
Clinical presentation				
- Asymptomatic			18 (58%)	
- Cardiac murmur			5 (16%)	
- Heart failure			6 (19.3%)	
- Prenatal suspicion			2 (6.4%)	
- Systemic hypertension			3 (9.7%)	
Baseline electrocardiographic anomalies (n°;%)			23 (74.1%)	n.a.
- Atrial enlargement			3 (9.6%)	
- LV hypertrophy/volume overload			14 (45%)	
- Right bundle branch block			7 (22.5%)	
- Ventricular pre-excitation			2 (6.4%)	
- Increased QTc interval			1 (1.8%)	
- Premature atrial complexes			1 (1.8%)	
Baseline echocardiographic findings (n°;%)				
- HCM			13 (42%)	
- HCM/NC			4 (13%)	
- DCM			8 (25.8%)	
- PAH			4 (13%)	
Major cardiac events (n°; %)			6 (19%)	
- Hospitalization due to HF			2 (6.4%)	
- Decease due to HF			2 (6.4%)	
- Resuscitated cardiac arrest			1 (1.8%)	
- PM implantation (high-grade AV block)			1 (2.8%)	
- Sudden cardiac death			1 (1.8%)	n.a.
Mortality	26 (30.2%)	12 (21.8%)	14 (45.1%)	< 0.01
- Cardiac cause			3 (9.6%)	
- Respiratory infection			2 (6.4%)	
- Metabolic crisis			2 (6.4%)	
- Stroke			1 (1.8%)	
- Not assessed			6 (19%)	

Abbreviations: *CVI* cardiovascular involvement; *LFUP* last follow-up; *LV* left ventricle; *HCM* hypertrophic cardiomyopathy; *NC* non-compaction; *DCM* dilative cardiomyopathy; *PAH* pulmonary hypertension; *HF* heart failure; *PM* pace-maker; *AV* atrioventricular

In 57 patients (66%) the diagnosis was confirmed by molecular analysis [Table 2]. MELAS syndrome due to MTTL1 A3243G mutation was the most frequent finding (9%), followed by TMEM70 (6%), NARP and Barth syndrome (5% each). Isolated mutations in subunits of respiratory chain complexes were also documented, in MTND6 (*n* = 2), MTND1 (n = 2) and MTND2, MTND3, MTND5, MTCYB1, MT-CO1 or MT-TC (*n* = 1 for each). Two patients were diagnosed with Kearns-Sayre disease, one with Sengers syndrome. A child showed clinical and genetic features consistent with Leber’s hereditary optic neuropathy. Large mt-DNA depletion not consistent with a specific syndrome was documented in two children. Less common mutations were found in POLG, ACAD9, SURF1, NDFUS1, SDHB, ELAC2, DGUOK and TK2 genes. Finally, 6 nuclear mutations (TIMMDC1, NUBPL, SFXN4, ATAD3A, SLC19A3, DARS2) were identified through whole-exome sequencing.

### Extra-cardiac involvement

Neurologic manifestations were the dominating features (52/86; 60%), mainly presenting with neurodevelopmental delay (36; 42%), epilepsy (28; 32.5%), hypo- or hypertonia (17; 19.7%), recurrent headache/cerebral stroke (6; 7%) and/or neuro-radiological features consistent with Leigh syndrome (5; 5.8%). No significant differences were documented between cardiac and non-cardiac cohorts (p 0.09). Other recurrent manifestations included myopathy (32%) and ocular involvement (19%). Hearing loss was documented in 8 patients, renal failure in 6 and diabetes in 5 [Table 2].

### Cardiovascular involvement

CVI was present in 31 patients (36%), with a significant male prevalence (M/F: 1.8; *p* < 0.01) and a mean age at onset of 5.7 ± 7.8 years (Table 1). In over one-third of patients (12/31; 39%) early CVI could be documented in the neonatal period (< 30 days); the onset of cardiomyopathy developed before 12 months of age in 14 children (45%) and before 5 years in 24 (67,7%). In 58% (18/31) ECG and/or echocardiographic abnormalities were identified in a pre-symptomatic phase, by routine screening following PMD diagnosis. In two additional patients (6%) cardiac hypertrophy was suspected prenatally, and confirmed after birth. CVI constituted the first presenting sign of metabolic disease in 13 (42%) of patients: heart failure was the most common clinical presentation (6/31; 19%), followed by cardiac murmur at clinical examination (5/31; 16%).

CVI was confirmed in all patients diagnosed with TMEM70 deficiency and Barth syndrome; MELAS syndrome was associated with heart disease in 62% of the children and NARP in 25%. Among patients with CI, mutations in ELAC2, NDUFS1, ACAD9, tRNA (Glu), MT-ND1 and MT-ND3 were individually documented in one cardiac patient each (3.2%). In addition one patient was diagnosed with Kearns Sayre disease, one with Sengers syndrome and another one presented with large mt-DNA deletion (Table 1).

Cardioactive treatment was prescribed in 24 (77%) of the 31 patients with CVI. Fourteen (45%) received beta-blockers (propranolol or metoprolol); 10 (32%) required chronic treatment with diuretics (furosemide and spironolactone); 8 (26%) were treated with Ace-inhibitors (captopril, enalapril), combined with angiotensin II receptor antagonists in two cases.

### ECG findings

ECG abnormalities were documented in 23 of the 31 patients (74%), 95% of them showing abnormal echo findings at first evaluation or through the follow-up. In 14 (45%) signs of ventricular hypertrophy were present at baseline evaluation; right bundle branch block and non-specific intra-ventricular delay were common (22%). Three patients (10%) met the criteria for right atrial enlargement (P pulmonale). Ventricular pre-excitation was detected in 2 children (6%); first-degree AV block, prolonged QTc interval and premature atrial complexes were less common (1 patient each). During follow-up, 2 patients developed premature ventricular complexes with moderate prevalence (VPCs < 10,000/day), which did not require medical treatment. Two patients developed complex ventricular arrhythmias, including one requiring ICD implantation in primary prevention. The child with Kearn-Sayre syndrome developed third degree AV block requiring permanent pacing.

### Echocardiographic findings

**Fig. 1:**
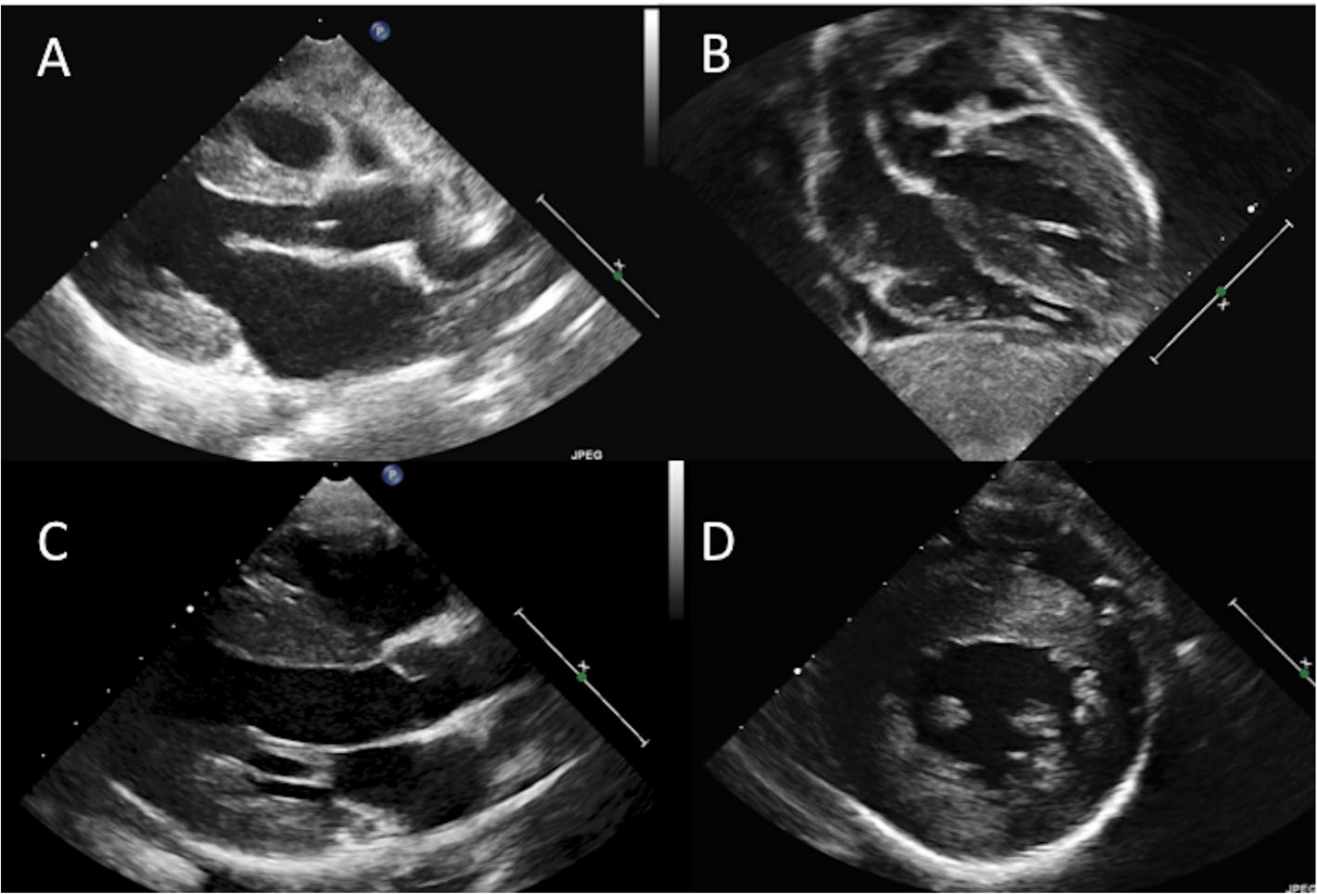
Hypertrophic cardiomyopathy with non-obstructive, concentric phenotype in a 6-month old female diagnosed with TMEM70 deficiency (Panel **a**: parasternal long-axis view, Panel **b**: sub-costal view); and in a 5-year old male with combined respiratory chain enzyme deficiencies (complex I-III-IV): Panel **c** (long-axis view) and **d** (parasternal short-axis view). All images are in diastolic phase

Hypertrophic cardiomyopathy was the most common phenotype (17/31; 58%); mainly in a non-obstructive form [Fig. 1]; dynamic obstruction was detected in a single patient affected by MELAS syndrome. Hypertrophy was concentric in 14 of the 17 patients, extending to the right ventricle in two. Asymmetric hypertrophy limited to interventricular septum was considerably less common (3/17; 17%). Notably, a mixed phenotype with aspects of ventricular non-compaction (HCM/NC) occurred in 4 children (24%). In 7 (23%) the phenotype was dilated cardiomyopathy; one additional patient presented with mild diastolic dysfunction and dilatation of the ascending aorta. Four patients (13%) presented with pulmonary artery hypertension. Arterial hypertension was documented at 24-h monitoring in 3 patients (10%).

### Long-term course and mortality

**Fig. 2:**
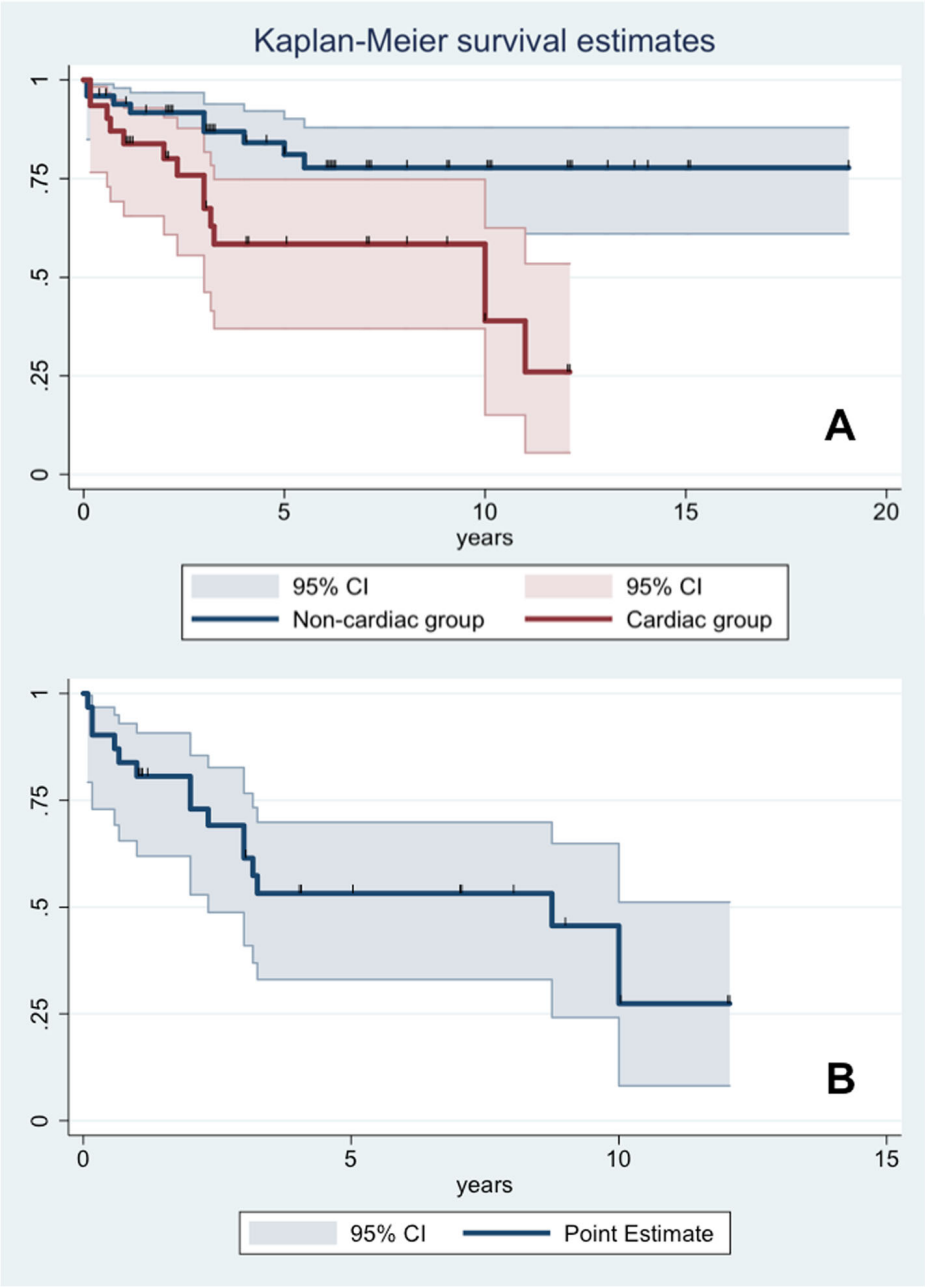
Kaplan Meier survival analysis (**a**) showing impaired survival (due to all-cause mortality) in patients with cardiovascular involvement (red line) and without CVI involvement (blue line) (*p* < 0.01). Time zero is defined as time at diagnosis of metabolic disorder. The bends display the 95% confidence interval (CI). A second curve (**b**) concerning MACE-free survival limited to the subgroup of CVI patients is reported in the bottom

Mean follow-up duration was 5.4 ± 4.3 years. Stable echocardiographic parameters (parietal and septal thickness, chamber volumes and biventricular contractility) were observed in most patients with CVI (14/17; 82%). However, compared to the group with normal cardiac findings, patients with CVI showed significantly worse prognosis (*p* < 0.01) [Fig. 2]: all-cause mortality was 45% (14/31) in the CVI versus 22% (12/55) in the non-CVI group. Mean age at death was 6.7 ± 8.2 years and 6.6 ± 5.7 years respectively (*p* = 0.8).

Major adverse cardiac events were recorded in six patients (6/31; 19%), including death due to end-stage heart failure (*n* = 2), sudden death due to ventricular fibrillation (*n* = 1), hospitalization due to acute heart failure (*n* = 2) and high-grade ventricular block requiring pace-maker implantation (*n* = 1).

**Fig. 3:**
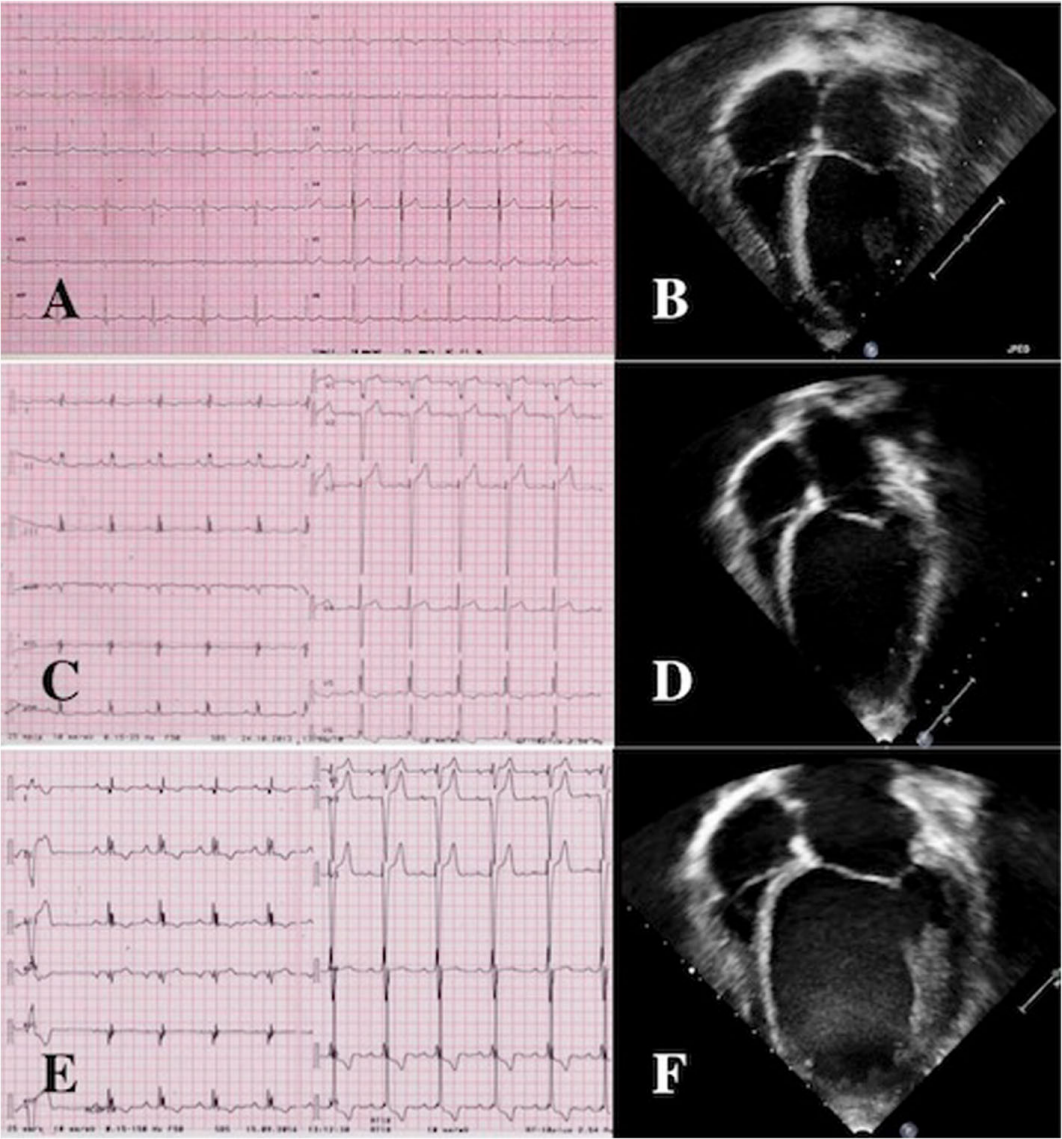
ECG end echocardiogram from a 16-year old patient diagnosed with ELAC2 mutation. Initial phenotype with mild, concentric left ventricular hypertrophy (Panels **a-b**) evolving to an intermediate (Panels **c-d**) and severe dilated/hypokinetic stage (Panels **e-f**)

Among the children presenting with HCM phenotype, adverse remodeling with evolution towards dilated phenotype was documented in a single patient diagnosed with ELAC2 mutation [Fig. 3]. The clinical course was complicated with sustained ventricular arrhythmias requiring ICD implantation. He was put on a cardiac transplantation list; however, he died shortly after due to recurrent ventricular fibrillation, despite appropriate ICD interventions. A second patient with Complex I deficiency and mixed HCM/NC cardiomyopathy developed progressive LV impairment and died due to refractory heart failure. Moderate depression in LVEF (40%) was recognized in a MELAS patient in the course of pneumonia; the child died within few weeks due to respiratory failure.

Among the 7 children with a dilative phenotype, 4 only had mild and stable LV dysfunction (FE 45–50%). Two children diagnosed with Barth syndrome showed a satisfactory recovery in LV function (EF from 25 to 50%) after cardioactive (ace-inhibitors, beta-blockers). Progressive systolic impairment was documented in a single patient with Senger syndrome; despite maximized pharmacologic treatment, the child died due to refractory heart failure.

Three of the 4 patients developing pulmonary hypertension died during follow-up: they were affected by MELAS, TMEM70 and tRNAGlu mutation.

Non-cardiac cause of death were registered in 6 patients; 3 died due to acute respiratory infection (2 diagnosed with MELAS, 1 with tRNAGlu mutation), 2 patients due to acute metabolic crisis, embodying the end-stage of the disease (MT-ND3) or exacerbated by infection (TMEM70); one patient (7.1%) affected by MELAS syndrome died as complication of cerebral stroke.

### Predictors of outcome

Early age at onset of cardiovascular symptoms was associated with adverse prognosis and premature death (*p* = 0.01). Age at presentation of CVI < 5 years constituted a negative predictor of outcome (*p* = 0.02); of note, each month delay in clinical onset was associated to a 2% reduction in the risk of mortality during childhood (*p* < 0.01). Finally, clinical onset characterized by acute heart failure led to an increased mortality risk (*p* = 0.04), irrespective of the age at presentation. Despite being more common in males, females exhibited a worse outcome with higher mortality rate (p 0.03). No gender-related differences were documented in terms of age at presentation (*p* = 0.2) of prevalence of heart failure at presentation (*p* = 0.6).

## Discussion

We investigated clinical profile and outcome of CVI in a cohort of 86 paediatric patients diagnosed with primary mitochondrial disorder. CVI was observed in 36% of our cohort, mainly in the form of hypertrophic, non-compaction or dilated cardiomyopathies and pulmonary hypertension. TMEM70 and Barth syndrome were systematically associated with CVI, but prevalence also proved high in MELAS syndrome. CVI was the presenting manifestation of the underlying metabolic disease in more than 40% of patients. Notably, we found a higher prevalence of early CVI than previous findings [5, 6, 12–14]: almost 40% of our children were diagnosed with cardiovascular disease within the first month of life (prenatally in 2 instances), half of them detected in a pre-symptomatic phase. Such high prevalence may have been elicited by the availability of a highly specialized centre for metabolic disorders, triggering early referral.

A timely diagnosis of PDM-related cardiovascular disease, especially in a pre-symptomatic phase, has relevant prognostic implications. Patients developing CVI exhibited a > 2-fold higher mortality rate compared to the counterpart without CVI (45% vs 22% respectively). Females, patients with diagnosis < 5 years and those with heart failure symptoms at presentation were at greatest risk. All these features identify individuals requiring aggressive surveillance and management of cardiovascular complications. In contrast with the relatively low prevalence of events in adults with PMD [15], MACEs were common in our children, occurring in almost 20%. Both heart failure-related and arrhythmic deaths occurred; among non lethal events, acute heart failure and advanced AV blocks represented treatable complications.

Our findings are in agreement with prior reports highlighting the adverse prognostic role of PMD-related CVI, suggesting a particularly unfavourable outcome in paediatric cohorts [6, 14, 16, 17]. The present study supports the view that CVI may act as the main driver of adverse outcome in PMD, rather than simply representing a marker of more aggressive multi-organ disease. Yet, it is difficult and probably wrong to generalize such conclusion to all PMD patients, given their vast and heterogeneous genetic spectrum. For example, the prognostic role of CVI appeared to be particularly relevant in distinct PMD syndrome, as ELAC2 and Sengers syndrome, in which refractory heart failure and arrhythmic act as primary cause of demise, independent of the degree of extra-cardiac involvement. This is in line with published case series [18–20], suggesting the opportunity of intensive treatment in this subset, including ventricular mechanical assistance and heart transplantation.

However, in other settings such as MELAS syndrome [8], patients presenting with CVI were globally more compromised, and heart disease seemed to represent a hallmark of phenotype severity rather than the primary cause of demise. This paradigm also seems to apply to TMEM70 deficiency, in which heart involvement is a constant feature but per se a stable one; acute decompensation may develop as consequence of metabolic crisis or severe infections [21]. Prompt treatment of metabolic crisis, appropriate nutrition with avoidance of fasting and prevention of infections (i.e. vaccination) represent the best “cardiac” preventive measures in these children. Likewise, in patients with Barth syndrome, survival rates have dramatically changed after the introduction of cardioactive treatment as well as prophylaxis for infections [22, 23] and stable recovery of LV function was observed in our cohort. Thus, individualized assessment and follow-up strategies based on genotype may represent the future in PMD case.

Mitochondrial disorders may be transmitted either as Mendelian trait or follow a matrilineal inheritance pattern; therefore, except for X-linked disorders (i.e. Barth syndrome), no gender prevalence is expected. As element of interest, we reported higher prevalence of CVI in males, but worse outcome in females (*p* = 0.03), similar to what has been reported for sarcomeric HCM in adult patients [24]. Whether and to what extent this gender bias entails social, as well as genetic, causes remains unclear. In our experience, poor social status and levels of educations are associated with delay in the identification of signs of infection, inadequate feeding and cardiac and metabolic decompensation in children, representing urgent unmet needs in paediatric healthcare.

Our data are consistent with previous literature reporting complex I deficiency as one of the most common enzyme defect in childhood mitochondrial disorders, without significant differences between the two groups of patients. The molecular basis of this phenomenon is difficult to assess, possibly related to its dual genetic control (nuclear and mtDNA) and to the fact that it is the largest complex in the mitochondrial respiratory chain. The clinical spectrum of complex I deficiency is heterogeneous but the majority of affected individuals show an early-onset, rapidly progressive disease, occurring with severe lactic acidosis, Leigh syndrome, leukoencephalopathy, hepatopathy and/or cardiomyopathy [25–27].

Treatment options in PMD patients remain limited. Shared protocols for pharmacologic treatment of paediatric cardiomyopathies and paediatric heart failure are not yet available; so far, current treatment strategies are commonly derived from adult guidelines and/or rely on the experiences of single centres. Non-pharmacologic options as mechanical ventricular assistance devices or heart transplantation are often considered unsuitable for patients affected by mitochondrial disorders, due to the concomitant presence of severe multi-organ involvement; however, this may not always be the case and the field deserves appropriate investigation. Heart transplantation is feasible, but candidate selection remains very challenging [28–32].

### Limitations

The present study has been conducted as retrospective research. Certain information as biochemical markers (NT-ProBNP, Troponins), mt-DNA heteroplasmy or instrumental data (systematic evaluation of diastolic function, cardiac magnetic resonance) were not available in all patients, despite systematic implementation of diagnostic protocols over the years. We could not assess a significant correlation between distinct pathogenic mutations, specific respiratory chain enzyme deficiency or echocardiographic phenotype and cardiac outcome; theses result may be partially explained by the heterogeneity of molecular diagnosis in our paediatric cohort and consequent small number of patients per molecular diagnosis, affecting statistical power. Larger studies base on international consortia are needed to confront this issue. Finally, the absence of standard treatment protocols for the management of PMD-related cardiac disorders, as well as concomitant metabolic treatment, did not allow a reliable estimate of therapeutic efficacy in our PMD patients.

## Conclusions

Cardiovascular involvement is a common feature of primary mitochondrial disorders and occurs earlier than previously described, across the PMD paediatric spectrum. CVI is associated with unfavourable prognosis and major adverse cardiac events are not rare in affected patients; however, there is ample variability reflecting the diverse genetic substrates. Female sex, acute heart failure and age at onset < 5 years are the main predictors of outcome associated with CVI. Cardiac assessment is strongly recommended in all patients with confirmed or suspected PMD, even if asymptomatic at the time of diagnosis, and regular cardiac follow-up is required. Moreover, our data suggest the need for tailored management of heart failure and sudden death prevention in children with PMD.

## Data Availability

The datasets used and/or analysed during the current study are available from the corresponding author on reasonable request.

## Abbreviations

PMD: Primary mitochondrial disorders
CVI: Cardiovascular involvement
MELAS: Mitochondrial encephalopathy, lactic acidosis, and stroke-like episodes syndrome
ICD: Implantable cardioverter defibrillator
PM: Pace-maker
HCM: Hypertrophic cardiomyopathy
DCM: Dilated cardiomyopathy
NC: Non-compaction
PAH: Pulmonary artery hypertension
AV: Atrio-ventricular
LV: Left ventricular
MACE: Major adverse cardiac events

## References

[1] Murphy E, Ardehali H, Balaban RS, DiLisa F, Dorn GW, Kitsis RN, Otsu K, Ping P, Rizzuto R, Sack MN, Wallace D, Youle RJ (2016). American Heart Association Council on basic cardiovascular sciences, council on clinical cardiology, and council on functional genomics and translational biology. Mitochondrial function, biology, and role in disease. A scientific statement from the American Heart Association. Circ Res.

[2] Fang EF, Scheibye-Knudsen M, Chua KF, Mattson MP, Croteau DL, Bohr VA (2016). Nuclear DNA damage signalling to mitochondria in ageing. Nat Rev Mol Cell Biol.

[3] Gong G, Song M, Csordas G, Kelly DP, Matkovich SJ, Dorn GW (2015). Parkin-mediated Mitophagy Directs Perinatal Cardiac Metabolic Maturation in Mice. Science.

[4] Lou G, Palikaras K, Lautrup S, Scheibye-Knudsen M, Tavernarakis N, Fang EF (2020). Mitophagy and Neuroprotection. Trends Mol Med.

[5] Holmgren D, Wahlandera H, Eriksson BO, Oldfors A, Holme E, Tulinius M (2003). Cardiomyopathy in children with mitochondrial disease: clinical course and cardiological findings. Eur Heart J.

[6] Scaglia F, Towbin JA, Craigen WJ, Belmont JW, O'Brian Smith E, Neish SR, Ware SM, Hunter JV, Fernbach SD, Vladutiu GD, Wong LJC, Vogel H (2004). Clinical Spectrum, morbidity, and mortality in 113 pediatric patients with mitochondrial disease. Pediatrics.

[7] Bernier FP, Boneh A, Dennett X, Chow CW, Cleary MA, Thorburn DR (2002). Diagnostic criteria for respiratory chain disorders in adults and children. Neurology.

[8] Brambilla A, Favilli S, Olivotto I, Calabri GB, Porcedda G, De Simone L, Procopio E, Pasquini E, Donati MA (2019). Clinical profile and outcome of cardiac involvement in MELAS syndrome. Int J Cardiol.

[9] Harel T, Yoon WH, Garone C, Gu S, Coban-Akdemir Z, Eldomery MK, Posey JE, Jhangiani SN, Rosenfeld JA, Cho MT, Fox S, Withers M, Brooks SM, Chiang T, Duraine L, Erdin S, Yuan B, Shao Y, Moussallem E, Lamperti C, Donati MA, Smith JD, HM ML, Eng CM, Walkiewicz M, Xia F, Pippucci T, Magini P, Seri M, Zeviani M, Hirano M, Hunter JV, Srour M, Zanigni S, Lewis RA, Muzny DM, Lotze TE, Boerwinkle E, Gibbs RA, Hickey SE, Graham BH, Yang Y, Buhas D, Martin DM, Potocki L, Graziano C, Bellen HJ, Lupski JR, Baylor-Hopkins Center for Mendelian Genomics; University of Washington Center for Mendelian Genomics (2016). Recurrent De Novo and Biallelic Variation of ATAD3A, Encoding a Mitochondrial Membrane Protein, Results in Distinct Neurological Syndromes. Am J Hum Genet.

[10] Kremer LS, Bader DM, Mertes C, Kopajtich R, Pichler G, Iuso A, Haack TB, Graf E, Schwarzmayr T, Terrile C, Koňaříková E, Repp B, Kastenmüller G, Adamski J, Lichtner P, Leonhardt C, Funalot B, Donati A, Tiranti V, Lombes A, Jardel C, Gläser D, Taylor RW, Ghezzi D, Mayr JA, Rötig A, Freisinger P, Distelmaier F, Strom TM, Meitinger T, Gagneur J, Prokisch H (2017). Genetic diagnosis of Mendelian disorders via RNA sequencing. Nat Commun.

[11] Hildick-Smith GJ, Cooney JD, Garone C, Kremer LS, Haack TB, Thon JN, Miyata N, Lieber DS, Calvo SE, Akman HO, Yien YY, Huston NC, Branco DS, Shah DI, Freedman ML, Koehler CM, Italiano JE, Merkenschlager A, Beblo S, Strom TM, Meitinger T, Freisinger P, Donati MA, Prokisch H, Mootha VK, DiMauro S, Paw BH (2013). Macrocytic anemia and mitochondriopathy resulting from a defect in sideroflexin 4. Am J Hum Genet.

[12] Garcia-Cazorla A, De Lonlay P, Nassogne MC, Rustin P, Touati G, Saudubray JM (2005). Long-term follow-up of neonatal mitochondrial cytopathies: a study of 57 patients. Pediatrics.

[13] Gibson K, Halliday JL, Kirby DM, Yaplito-Lee J, Thorburn DR, Boneh. Mitochondrial oxidative phosphorylation disorders presenting in neonates: clinical manifestations and enzymatic and molecular diagnoses. Pediatrics 2008;122:1003–1008. 10.1542/peds.2007-3502 18977979

[14] Schiff M, Ogier de Baulny H, Lombèsd A (2011). Neonatal cardiomyopathies and metabolic crises due to oxidative phosphorylation defects. Sem Fet Neonat Med.

[15] Wahbi K, Bougouin W, Béhin A, Stojkovic T, Bécane HM, Jardel C, Berber N, Mochel F, Lombès A, Eymard B, Duboc D, Laforêt P (2015). Long-term cardiac prognosis and risk stratification in 260 adults presenting with mitochondrial diseases. Eur Heart J.

[16] Böhm M, Pronicka E, Karczmarewicz E, Pronicki M, Piekutowska-Abramczuk D, Sykut-Cegielska J, Mierzewska H, Hansikova H, Vesela K, Tesarova M, Houstkova H, Houstek J, Zeman J (2015). Retrospective, multicentric study of 180 children with cytochrome c oxidase deficiency. Pediatr Res.

[17] Lev D, Nissenkorn A, Leshinsky-Silver E, Sadeh M, Zeharia A, Garty BZ, Blieden L, Barash V, Lerman-Sagie T (2004). Clinical presentations of mitochondrial cardiomyopathies. Pediatr Cardiol.

[18] Haghighi A, Haack TB, Atiq M, Mottaghi H, Haghighi-Kakhki H, Bashir RA, Ahting U, Feichtinger RG, Mayr JA, Rötig A, Lebre AS, Klopstock T, Dworschak A, Pulido N, Saeed MA, Saleh-Gohari N, Holzerova E, Chinnery PF, Taylor RW, Prokisch H (2014). Sengers syndrome: six novel AGK mutations in seven new families and review of the phenotypic and mutational spectrum of 29 patients. Orphanet J Rare Dis.

[19] Haack TB, Kopajtich R, Freisinger P, Wieland T, Rorbach J, Nicholls TJ, Baruffini E, Walther A, Danhauser K, Zimmermann FA, Husain RA, Schum J, Mundy H, Ferrero I, Strom TM, Meitinger T, Taylor RW, Minczuk M, Mayr JA, Prokisch H (2013). ELAC2 mutations cause a mitochondrial RNA processing defect associated with hypertrophic cardiomyopathy. Am J Hum Genet.

[20] Shinwari ZMA, Almesned A, Alakhfash A, Al-Rashdan AM, Faqeih E, Al-Humaidi Z, Alomrani A, Alghamdi M, Colak D, Alwadai A, Rababh M, Al-Fayyadh M, Al-Hassnan ZN (2017). The phenotype and outcome of infantile cardiomyopathy caused by a homozygous ELAC2 mutation. Cardiology.

[21] Magner M, Dvorakova V, Tesarova M, Mazurova S, Hansikova H, Zahorec M, Brennerova K, Bzduch V, Spiegel R, Horovitz Y, Mandel H, Eminoğlu FT, Mayr JA, Koch J, Martinelli D, Bertini E, Konstantopoulou V, Smet J, Rahman S, Broomfield A, Stojanović V, Dionisi-Vici C, van Coster R, Morava E, Sperl W, Zeman J, Honzik T (2015). TMEM70 deficiency: long-term outcome of 48 patients. J Inherit Metab Dis.

[22] Rigaud C, Lebre AS, Touraine R, Beaupain B, Ottolenghi C, Chabli A, Ansquer H, Ozsahin H, Di Filippo S, De Lonlay P, Borm B, Rivier F, Vaillant MC, Mathieu-Dramard M, Goldenberg A, Viot G, Charron P, Rio M, Bonnet D, Donadieu J (2013). Natural history of Barth syndrome: a national cohort study of 22 patients. Orphanet J Rare Dis.

[23] Reynolds S (2015). Successful management of Barth syndrome: a systematic review highlighting the importance of a flexible and multidisciplinary approach. J Multidiscip Healthc.

[24] Olivotto I, Maron MS, Adabag AS, Casey SA, Vargiu D, Link MS, Udelson JE, Cecchi F, Maron BJ (2005). Gender-related differences in the clinical presentation and outcome of hypertrophic cardiomyopathy. J Am Coll Cardiol.

[25] Fassone E, Rahman S (2012). Complex I deficiency: clinical features, biochemistry and molecular genetics. J Med Genet.

[26] Swalwell H, Kirby DM, Blakely EL, Mitchell A, Salemi R, Sugiana C, Compton AG, Tucker EJ, Ke BX, Lamont PJ, Turnbull DM, McFarland R, Taylor RW, Thorburn DR (2011). Respiratory chain complex I deficiency caused by mitochondrial DNA mutations. Eur J Hum Genet.

[27] Danhelovska T, Kolarova H, Zeman J, Hansikova H, Vaneckova M, Lambert L (2020). Multisystem mitochondrial diseases due to mutations in mtDNA-encoded subunits of complex I. BMC Pediatr.

[28] Parikh S, Karaa A, Goldstein A, Ng YS, Gorman G, Feigenbaum A, Christodoulou J, Haas R, Tarnopolsky M, Cohen BK, Dimmock D, Feyma T, Koenig MK, Mundy H, Niyazov D, Saneto RP, Wainwright MS, Wusthoff C, McFarland R, Scaglia F (2016). Solid organ transplantation in primary mitochondrial disease: proceed with caution. Mol Genet Metab.

[29] Limongelli G, Tome-Esteban M, Dejthevaporn C, Rahman S, Hanna MG, Elliott PM (2010). Prevalence and natural history of heart disease in adults with primary mitochondrial respiratory chain disease. Eur J Heart Fail.

[30] Bates MG, Nesbitt V, Kirk R, He L, Blakely EL, Alston CL, Brodlie M, Hasan A, Taylor RW, McFarland R (2012). Mitochondrial respiratory chain disease in children undergoing cardiac transplantation: a prospective study. Int J Cardiol.

[31] Schmauss D, Sodian R, Klopstock T, Deutsch MA, Kaczmarek I, Roemer U, Reichart B, Daebritz SH (2007). Cardiac transplantation in a 14-yr-old patient with mitochondrial encephalomyopathy. Pediatr Transplant.

[32] Santorelli FM, Gagliardi MG, Dionisi-Vici C, Parisi F, Tessa A, Carrozzo R, Piemonte F, Pfeiffer K, Schägger H, Bertini E (2002). Hypertrophic cardiomyopathy and mtDNA depletion. Successful treatment with heart transplantation. Neuromuscul Disord.

